# Prediction of Breeding Values for Dairy Cattle Using Artificial Neural Networks and Neuro-Fuzzy Systems

**DOI:** 10.1155/2012/127130

**Published:** 2012-09-09

**Authors:** Saleh Shahinfar, Hassan Mehrabani-Yeganeh, Caro Lucas, Ahmad Kalhor, Majid Kazemian, Kent A. Weigel

**Affiliations:** ^1^Department of Animal Science, University College of Agriculture and Natural Resources, University of Tehran, Karaj, Iran; ^2^Center of Excellence: Control and Intelligent Processing, School of Electrical and Computer Engineering, University of Tehran, Iran; ^3^Department of Dairy Science, University of Wisconsin-Madison, Madison, WI 53706, USA

## Abstract

Developing machine learning and soft computing techniques has provided many opportunities for researchers to establish new analytical methods in different areas of science. The objective of this study is to investigate the potential of two types of intelligent learning methods, artificial neural networks and neuro-fuzzy systems, in order to estimate breeding values (EBV) of Iranian dairy cattle. Initially, the breeding values of lactating Holstein cows for milk and fat yield were estimated using conventional best linear unbiased prediction (BLUP) with an animal model. Once that was established, a multilayer perceptron was used to build ANN to predict breeding values from the performance data of selection candidates. Subsequently, fuzzy logic was used to form an NFS, a hybrid intelligent system that was implemented via a local linear model tree algorithm. For milk yield the correlations between EBV and EBV predicted by the ANN and NFS were 0.92 and 0.93, respectively. Corresponding correlations for fat yield were 0.93 and 0.93, respectively. Correlations between multitrait predictions of EBVs for milk and fat yield when predicted simultaneously by ANN were 0.93 and 0.93, respectively, whereas corresponding correlations with reference EBV for multitrait NFS were 0.94 and 0.95, respectively, for milk and fat production.

## 1. Introduction

Machine learning techniques, such as decision trees and artificial neural networks (ANN), are used increasingly in agriculture, because they are quick, powerful, and flexible tools for classification and prediction applications, particularly those involving nonlinear systems [1]. These techniques have been used for detection of mastitis [2], detection of estrus [3], and discovery of reasons for culling [1]. Decision trees and related methods have also been used for analysis of lactation curves [4], interpretation of somatic cell count data [5], and assessment of the efficiency of reproductive management [6, 7]. In addition, ANN have been used for the prediction of total farm milk production [8], prediction of 305-day milk yield [9, 10], and detection of mastitis [11, 12]. 

Fuzzy logic, which involves classification of variables into fuzzy sets with degrees of membership between 0 and 1, has recently found its way into agricultural research [13, 14]. Applications have included development of decision-support systems for analyzing test-day milk yield data from Dairy Herd Improvement (DHI) programs [15]. Detection of mastitis and estrus from automated milking systems [16, 17], and definition of contemporary groups for the purpose of genetic evaluation [18]. A key challenge in the use of fuzzy sets is the development of appropriate membership functions (MF). Due to the relative simplicity of building ANN, these may be used to reduce the time and computational burden associated with MF determination. In fact, developments in neural network-driven fuzzy control suggest that these technologies may be quite complementary [19]. In multivariate prediction models, ANN could be used to develop MF for fuzzy sets from input variables such as milk yield, parity, or stage of lactation. Such tools have been used to develop decision-support software for culling and replacement decisions [20], as well as for qualitative assessment of milk production [21] in dairy cattle.

From a dairy cattle breeding viewpoint, accurate and timely prediction of lactation milk yield of progeny is a key prerequisite to selection of genetically superior males. In a breeding program, genetic progress can be maximized through accurate identification of superior animals that will be selected as parents of the next generation and therefore breeding goals can be achieved. A key component of this process is fast and reliable prediction of breeding values for selection candidates. However, prediction of breeding values is often a computationally challenging and time consuming task, and therefore it is undertaken only periodically (e.g., quarterly or semiannually) in most countries. Rapid, low-cost alternatives that can provide approximate predictions of breeding values with acceptable accuracy could allow more timely selection and culling decisions by breeding companies or dairy producers. Rapid identification of superior males can lead to earlier collection and distribution of semen and more rapid genetic progress [21, 22]. In several studies, back-propagation algorithms have been used to develop ANN for the prediction of 305-day milk, fat, and protein [21, 23]. However, there has not been any published research into the application of neuro-fuzzy networks and ANNs in the prediction of EBV in dairy cows. 

The objective of this current study was to investigate the potential of a hybrid intelligent system that combines artificial neural networks and fuzzy logic, also known as a neuro-fuzzy system (NFS) or neuro-fuzzy network (NFN) in order to compute the breeding values of Holstein cows for milk and fat production based on their performance data and EBV of their parents.

## 2. Material

### 2.1. Data Collection and Preprocessing

Data were provided by the Animal Breeding Center of Iran (ABCI, Tehran) and consisted of 119,899 lactation records of first parity Holstein cows that calved between 22 and 36 mo of age during the time period from 1990 to 2005. Milk and fat yield records were pre-adjusted for milking frequency. Data regarding environmental conditions included ambient temperature, ambient humidity, and length of the photoperiod. Herds included in the present study were representative of large commercial dairy herds in Iran. In Iran, milk production involves traditional dairy farms, which consist of roughly 6 million native and crossbred dairy cattle, and large commercial farms, which consist of approximately 0.8 million Holstein cows. Herds in the latter group practice intensive management and feed a total mixed ration containing concentrates (corn grain, soybean meal, fish meal, cotton seed, cottonseed meal, barley grain, canola meal, beet pulp, fat powder, vitamins, and minerals), alfalfa, and corn silage. These herds contain up to 3000 lactating cows, which are housed in free stalls and milked in parlor systems. Average milk production in these herds ranges from 8000 to 10,000 kg per 305 d lactation, and this exceeds the national average of about 6300 kg for Iranian Holsteins. Artificial insemination is used in more than 75% of the herds, and most cows are inseminated with imported semen or semen provided by ABCI. Generally speaking, ABCI is responsible for recording data, computing genetic evaluations, and establishing breeding strategies. Data regarding production traits (milk, fat, and protein yield), functional traits (longevity, calving ease, somatic cell count, and female fertility), and physical conformation traits are recorded on the majority of large commercial farms, and EBV for milk and fat yield and fat percentage are provided to farmers twice a year. 

### 2.2. Genetic Analysis

Data were prepared using Visual FoxPro v6.0 (Microsoft, Redmond, WA, USA). The following multiple-trait animal model for best linear unbiased prediction (BLUP) was used to compute EBV for milk and fat yield:

(1) $$\begin{array}{c} y_{ijkl}={\text{HYS}}_{i}+{\text{Age}}_{j}+{\text{DIM}}_{k}+a_{l}+e_{ijkl}, \end{array}$$

where *y*_*ijkl*_ = milk or fat yield observation on the *l*th animal in the *k*th level of days in milk, *j*th level of age at calving, and *i*th herd-year-season class, HYS_*i*_ = fixed effect of the *i*th herd-year-season class, Age_*j*_ = fixed effect of *j*th level of age at first calving, DIM_*k*_ = fixed effect of *k*th level of days in milk, *a*_*l*_ = random genetic effect of *l*th animal, distributed as **A** *σ*_*a*_^2^, and *e*_*ijkl*_ is a random residual effect, distributed as **I** *σ*_*e*_^2^. The genetic analysis was implemented using the PEST software [24]. In subsequent analyses using ANN and NFS, the sire and dam EBV from this genetic analysis were used as input variables, whereas the individual cow EBV were used as reference EBV for calculation of predictive ability of the networks.

### 2.3. Artificial Neural Networks

An artificial neural network, or ANN, is often simply referred to as a neural network, and it represents a nonlinear statistical modeling tool that is based on the concept of a biological neural network. Information flows through the network during the learning phase, and the ANN adapts its structure in order to model complex relationships between the input and output variables. The ANN consists of basic units, termed neurons, whose design is suggested by their biological counterparts. These artificial neurons have input paths, just as biological neurons have dendrites, and they have output paths, just as biological neurons have axons [9]. Both artificial and biological neurons also have predispositions (biases) that affect the strength of their output. The neuron combines the inputs, incorporates effects of the predisposition, and outputs signals. Learning occurs in both real and artificial neurons, and this alters the strength of connections between the neurons and the biases [25].

The training of ANN often facilitates discovery of previously unknown relationships between input and output variables, and these relationships have been used successfully in both classification and prediction problems [26]. Recognition of patterns in ANN occurs through training with data samples and is independent of the particular form of the information [27]. However, the pattern recognition ability of networks can be improved by various techniques. Common approaches to improving network performance include: finding an optimum network architecture, determining an appropriate number of training cycles, varying the combinations of input variables [28], customizing the values of learning parameters [23], and preselecting or preprocessing of the data [27].

In this research, we used a feed forward backpropagation multilayer perceptron (MLP herein) algorithm. We used a four layer MLP containing 1 input layer, 2 hidden layers, and 1 output layer. Each node in the input layer corresponds to one explanatory variable. Nodes in the hidden layer contain hyperbolic tangent activation functions [29], *h* = (*e* ^*y*_*i*_^ − *e* ^−*y*_*i*_^)/(*e* ^*y*_*i*_^ + *e* ^−*y*_*i*_^) and they take a weighted sum of all input variables, *y*_*i*_ = ∑_*j*_ *ω*_*ji*_ *χ*_*i*_, where *χ*_*i*_ is an input variable and *ω*_*ji*_ is corresponding weight in layer *j*. Similarly, the output node(s) take a weighted sum of all nodes in second hidden layer and use the same activation function to calculate the output value. Learning (updating weights) in the backpropagation algorithm starts by summing the errors over all of the network output unit(s). For each output unit *k*, the error term is *E*_*k*_ = *o*_*k*_(1 − *o*_*k*_)  (*t*_*k*_ − *o*_*k*_), where *t*_*k*_ and *o*_*k*_ are target and output for *k*th output of *d*th training example, respectively. Then, for each hidden layer the error term will be *E*_*h*_ = *o*_*h*_(1 − *o*_*h*_)∑_*k*∈output_ *ω*_*kh*_ *E*_*k*_, where *o*_*h*_ is the output of the hidden layer and *ω*_*kh*_ is the weight of *k*th output neuron. Eventually, for updating each weight in the network we use *ω*_*ji*_ = *ω*_*ji*_ + Δ*ω*_*ji*_ whit Δ*ω*_*ji*_ = *ηE*_*j*_ *x*_*ji*_, where *η* is called the learning rate (e.g., 0.05), *E*_*j*_ is the error term for the *j*th node, and *x*_*ji*_ is the input value for *j*th node in *i*th layer to which the weight is applied [30].

The tangent hyperbolic function also ranges from −1 to 1 and is differentiable, which has two advantages. First, it is necessary when using in backpropagation algorithm and second it gives a prediction range between −1 and 1 which is well suited for this study, because in our case, breeding values can take both positive and negative values.

### 2.4. Fuzzy Logic

Fuzzy logic is a form of multivalued logic that deals with approximate (rather than precise) reasoning and multiple truth values (rather than simply true and false). It involves the use of fuzzy sets, comprised of various categories that are expressed qualitatively by an expert, to which an element could partially belong. The degree to which an element belongs to a fuzzy set is defined by a membership function, or MF. For example, the milk production records of individual cows could be classified by an expert as very low, low, medium, high or very high. These categories would be represented by five fuzzy sets, and the record of a specific cow might belong partially to each of two adjacent sets, such as very low and low. When using a 100% membership scale, the expert may infer that a milk production record of 7134 kg belongs 90% to the very low set and 10% to the low set [31]. The functions that define the degrees of membership for specific values of the independent variable (in this case, milk production) are known as membership functions.

### 2.5. Neuro-Fuzzy Systems

Neuro-fuzzy systems, or NFS, are hybrid intelligent systems that combine the subjective reasoning features of fuzzy logic with the learning structure of neural networks. As such, NFS represent fuzzy logic models that are partially designed from expert knowledge and partially learned from the data. The close linkage between fuzzy logic models and neural networks motivated this data-driven approach to fuzzy modeling. Typically, the fuzzy logic model is represented in the structure of a neural network, and machine learning methods that have been established in a neural network context are applied to the NFS. The contemporary viewpoint is that fuzzy models can be learned directly from data, without first being drawn in a neural network structure, and some learning methods that are applied have no relationship to ANN. Nevertheless, the original terms of fuzzy neural network and neuro-fuzzy system have persisted for all types of fuzzy models that are learned from data [32].

The fundamental approach with locally linear neuro-fuzzy models (LLNF) is to divide the input space into small linear subspaces with fuzzy validity functions [32, 33]. Each linear part, along with its validity function which is a normalized Gaussian function, can be described as a fuzzy neuron. Thus, the total model is a NFS with one hidden layer, and a linear neuron in the output layer that simply computes the weighted sum of the outputs of locally linear neurons. Global optimization of the linear consequent parameters is carried out by least-squares [32]. An incremental tree-based learning algorithm, known as the locally linear model tree (LOLIMOT), can be used to tune the rule premise parameters, that is, to determine the validation hypercube for each locally linear model. At each iteration, the worst-performing locally linear neuron is designated to be divided. This learning algorithm provides an optimal linear or nonlinear model with maximum generalization, and it performs well in prediction applications. Only one parameter, the embedding dimension, must be specified before implementing the algorithm.

**Figure 1:**
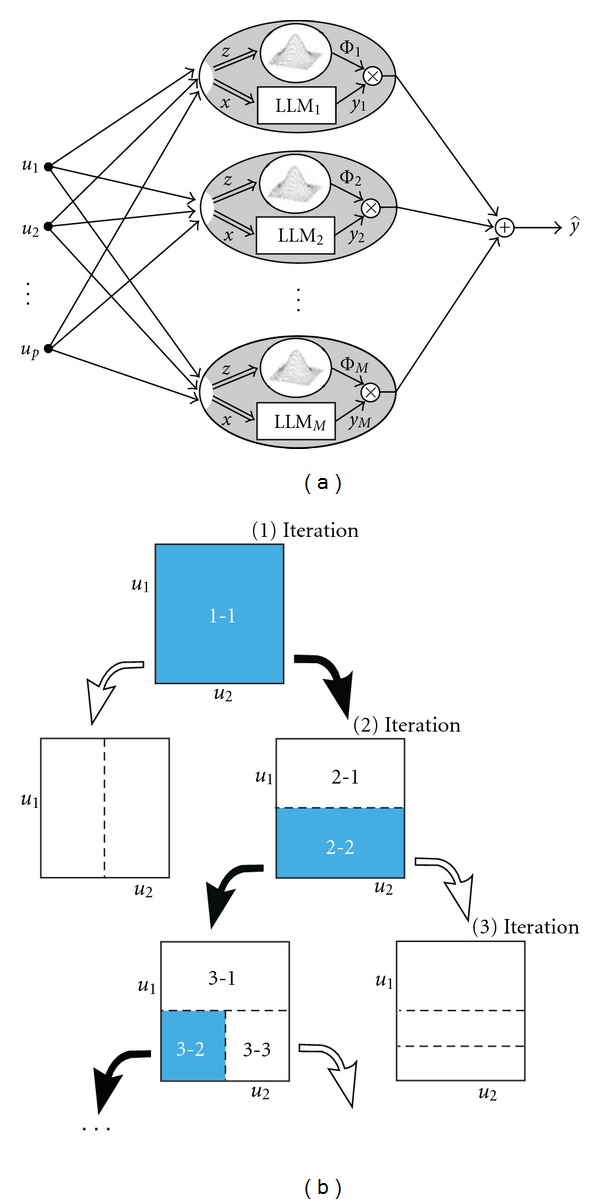
(a) Description of LOLIMOT Architecture, (b) description of LOLIMOT algorithm.

The structure and behavior of the local linear model tree algorithm is shown in Figures 1(a) and 1(b), respectively. The output of each local linear model is calculated by ${\hat{y}}_{i}=\omega_{i0}+\omega_{i1}u_{1}+\cdot \cdot \cdot +\omega_{ip}u_{p}$, where *u* = [*u*_1_, *u*_2_,…, *u*_*p*_] is the vector of inputs, *ω*_*i*_ s are linear coefficients, and a linear layer in the output simply calculates the weighted sum of each neuron (here local linear model outputs) as follows:

(2) $$\begin{array}{c} \hat{y}=\sum_{i=1}^{M}{\hat{y}}_{i}\phi_{i}\left(u\right), \end{array}$$

where *ϕ*_*i*_(*u*)  is the validity function of each neuron, calculated as *ϕ*_*i*_(*u*) = *μ*_*i*_(*u*)/∑_*j*=1_^*M*^ *μ*_*j*_(*u*)

(3) $$\begin{array}{c} \mu_{i}\left(u\right)=\exp\left\{-\frac{1}{2}\left[\frac{{\left(u_{i}-c_{i1}\right)}^{2}}{\sigma_{i1}^{2}}+\cdot \cdot \cdot +\frac{{\left(u_{p}-c_{p1}\right)}^{2}}{\sigma_{p1}^{2}}\right]\right\}\\ =\prod_{j=1}^{p}\exp\left[-\frac{1}{2}\frac{{\left(u_{j}-c_{ij}\right)}^{2}}{\sigma_{ij}^{2}}\right]. \end{array}$$

The best advantage of LOLIMOT is its low computational time, which is linear with respect to the number of fuzzy neurons [32].

### 2.6. Network Development and Error Criteria

In the implementation of ANN methodology for predicting EBV of Iranian Holstein cows for milk and fat yield, several ANN were designed using forward stepwise selection of input variables which means that we started with only one input variable (Milk 2x) and tried to predict EBV for milk. Then we kept adding other variables to the input vector until we reached the full set of available input variables, as shown in Table 1. Only the best-performing networks were selected based on RMSE and the correlations between predicted output and actual EBV and are discussed herein. This trial and error approach for “tuning” the network is necessary to determine the optimum structure and appropriate parameters. These networks, which were developed using the multilayer perceptron (MLP) algorithm, have two hidden layers and tangent hyperbolic activation functions. In the first hidden layer, the number of nodes was chosen to be twice the number of input variables, and in second hidden layer, the number of nodes was chosen to be equal to the number of inputs. Three learning rules were considered when training the networks: momentum, conjugate gradient [34], and Levenberg [35]. However, because results were nearly identical for all three learning rules, only results from the conjugate gradient approach are discussed herein. These algorithms were implemented using NeuroSolutions v5.0 (NeuroDimensions, Gainesville, FL, USA). The maximum number of epochs was set to 5000. Each network was trained five times with different initial random weights, and the best weight was chosen for testing each network. 

A “training set,” which consisted of a random sample of 7000 observations from the full data set, was used for initial development and each the network. Subsequently, a “tuning set,” which consisted of a random sample of 1000 additional observations, was used to optimize the structure of the network and determine appropriate parameter values via cross-validation. Finally, a “testing set,” which consisted of a random sample of 2000 independent observations, was used to validate the performance of the network via testing. The error criteria used to evaluate network performance included root mean square error (RMSE) and the correlation between predicted and reference EBV (*r*), where reference EBV correspond to animal model BLUP EBVs from the genetic analysis described in an earlier section:

(4) $$\begin{array}{c} \text{RMSE}=\sqrt{\frac{\sum_{i}{\left(x_{i}-d_{i}\right)}^{2}}{n}}\;\;\\ r=\frac{\sum_{i}\left(x_{i}-\overset{-}{x}\right)\left(d_{i}-\overset{-}{d}\right)/\left(n-1\right)}{\sqrt{\sum_{i}{\left(d_{i}-\overset{-}{d}\right)}^{2}/\left(n-1\right)\sqrt{\sum_{i}{\left(x_{i}-\overset{-}{x}\right)}^{2}/\left(n-1\right)}}}, \end{array}$$

where *n* = number of observations in the data set; *d*_*i*_ = reference output for observation *i*, with mean $\overset{-}{d}$; *x*_*i*_ = predicted output for observation *i*, with mean $\overset{-}{x}$. 

The data set described above was also used to train and evaluate neuro-fuzzy systems with the locally linear model tree algorithm, or LOLIMOT, using MATLAB v7.0 (The MathWorks, Natick, MA, USA). In these networks, prediction of desired outputs progressed until 50 locally linear models were developed. Subsequently, the best model was chosen according to the RMSE error criterion, and this model was used for validation of the network in the testing set. 

## 3. Results and Discussion

A total of 20 networks were evaluated for each multilayer perceptron and locally linear model tree algorithm. The input variables considered in each of the 20 networks are presented in Table 1, and these include: age at first calving, days in milk, ambient temperature, ambient humidity, length of the photoperiod, raw and adjusted (for milking frequency) milk, and fat production of each cow and the average of her contemporaries, and the milk and fat EBV of her parents. The output variables included: single-trait milk EBV of the cow (networks 1 to 13), single-trait fat EBV of the cow (networks 14 and 15), and multitrait milk and fat EBV of the cow (networks 16 to 20). 

### 3.1. Prediction of EBV for Milk Yield

In experiments 1 to 3, milk yield EBV were predicted as a single trait, using ANN via the MLP algorithm and NFS via the LOLIMOT algorithm as a function of fix effects, milk production, environmental factors and milk yield EBV of the dams. Results are given in Table 2. As the number of input variables increased in networks 1 to 3, the correlation between actual and predicted EBV increased, and the error criteria decreased, specially with regard to RMSE. In experiments 1 and 2, NFS had better performance than the ANN, whereas in experiment 3 performance of ANN was slightly superior. 

In experiments 4 to 6, milk yield EBV of the dams were not considered in the vector of input variables, but milk yield EBV of the sires were included. This resulted in a substantial decrease in the correlation between reference EBV and EBV predicted by the ANN and NFS, suggesting that EBV of the dam is a more useful variable for prediction of EBV of her daughters. This was an unexpected result given that sires' EBV are generally more accurate than dams' EBV, and it was most likely due to a common environmental component between a cow and her dam (note that herd-year-season was not included in the ANN and NFS because this variable would have had explanatory power in the training set, while providing no predictive power in the testing set, even for future observations in the same herds). 

In experiments 7 to 9, environmental variables were considered along with the EBV of both the cow's sire and the cow's dam. This resulted in higher correlations, as compared with experiments 1 to 3 and experiments 4 to 6, most notably the latter. This suggests that both sire and dam EBV can be useful predictors of the EBV of their offspring, as one would expect, but that the dam's EBV provides more information in this type of analysis for the reason noted previously. In experiments 7 and 8, correlations and RMSE criteria indicated greater predictive ability for NFS, whereas in experiment 9, predictive ability was slightly greater for ANN. 

In experiments 10 and 11, additional variables such as herd average, fat yield, humidity and length of day were included in the input vector. Predictive ability improved with inclusion of these variables; presumably most of this gain can be attributed to the inclusion of herd average. In experiment 11, fat yield EBV of the dam was also considered. Unexpectedly, addition of this variable improved performance of NFS slightly, but performance of ANN deteriorated, apparently because it introduced additional “noise” into the analysis. In experiments 12 and 13, milk yield EBV of the sire was also added to the input vector. In this case, inclusion of the dam's EBV for fat yield provided a very small increase in predictive ability of ANN and a very slight decrease in predictive ability of NFS, suggesting that dam's EBV for fat yield is largely redundant once milk yield EBV of the sire and dam and milk and fat yield of the cow have already been considered. 

Overall, the predictive ability of the best networks for milk yield, namely, experiment 13 for ANN and experiment 12 for NFS, was outstanding. Correlations between predicted milk yield EBV from the ANN and NFS analyses and reference EBV from BLUP analysis of the full data set were 0.92 and 0.93, respectively. 

### 3.2. Prediction of EBV for Fat Yield

In experiments 14 and 15, the objective was to predict fat yield EBV in a single trait analysis. As shown in Table 1, input variables were equivalent to those used for prediction of milk yield EBV in experiments 12 and 13 with fat EBV of Dam and Sire replacing milk EBV of Dam and Sire in those experiments. However, as shown in Table 3, performance was slightly better for prediction of EBV for fat than for milk, with correlations between ANN and NFS predictions and reference EBVs of 0.93 and 0.93, respectively, in experiment 15. 

### 3.3. Simultaneous Prediction of Milk and Fat EBV

In experiments 16 to 20, the objective was to jointly predict EBVs for milk yield and fat yield in a single analysis. Total milk yield of the animal (i.e., beyond 305 d) was included as an input variable in experiments 17, 19, and 20, and herd average for total milk yield was also included in experiment 20. As shown in Table 4, the addition of total milk yield provided a slight improvement in predictive ability in the NFS, but performance of the ANN deteriorated slightly, perhaps indicating that the information provided by this variable was largely redundant. Experiment 18 was equivalent to the single-trait analyses (i.e., its input variables was union set of variables in experiments 13 and 15), and predictive ability was equal to or better than in the single-trait analyses. In both experiments 18 and 19, performance of the NFS was superior to that of the ANN. Lastly, in experiment 20, all available explanatory variables listed in Table 1 were used in the simultaneous prediction of EBV for milk and fat yield. As one might expect, this experiment provided the highest correlations with reference EBV and, in general, the smallest RMSE of prediction. 

**Figure 2:**
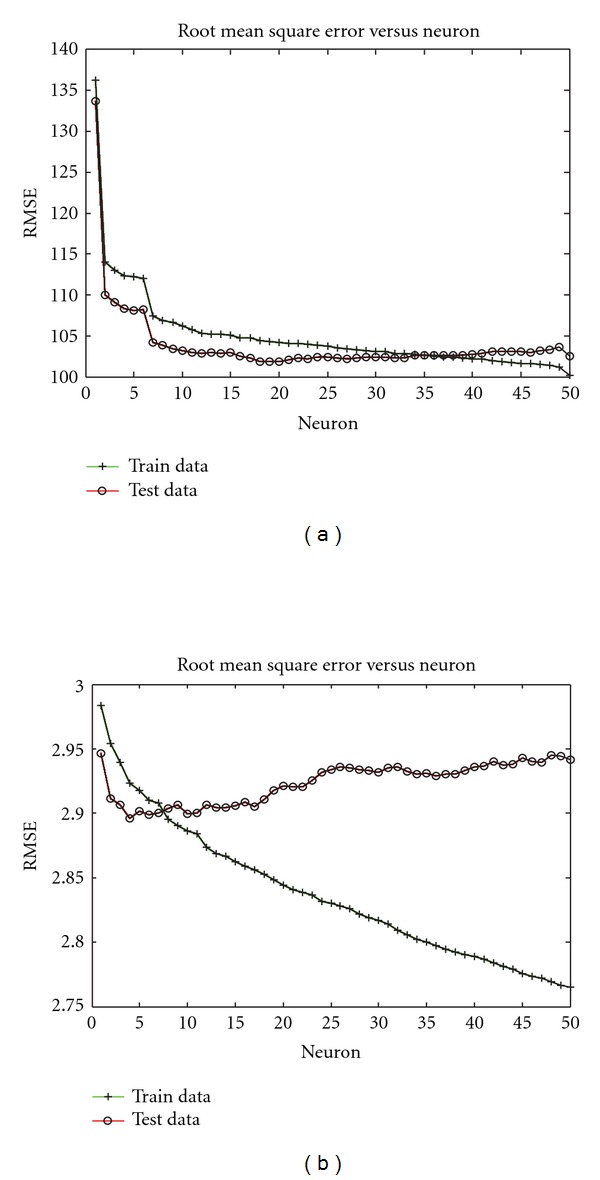
Root mean square error (RMSE) as a function of number of neurons in the single-trait neuro-fuzzy models: (a) prediction of milk yield EBV in experiment 12, and (b) prediction of fat yield EBV in experiment 15.

**Figure 3:**
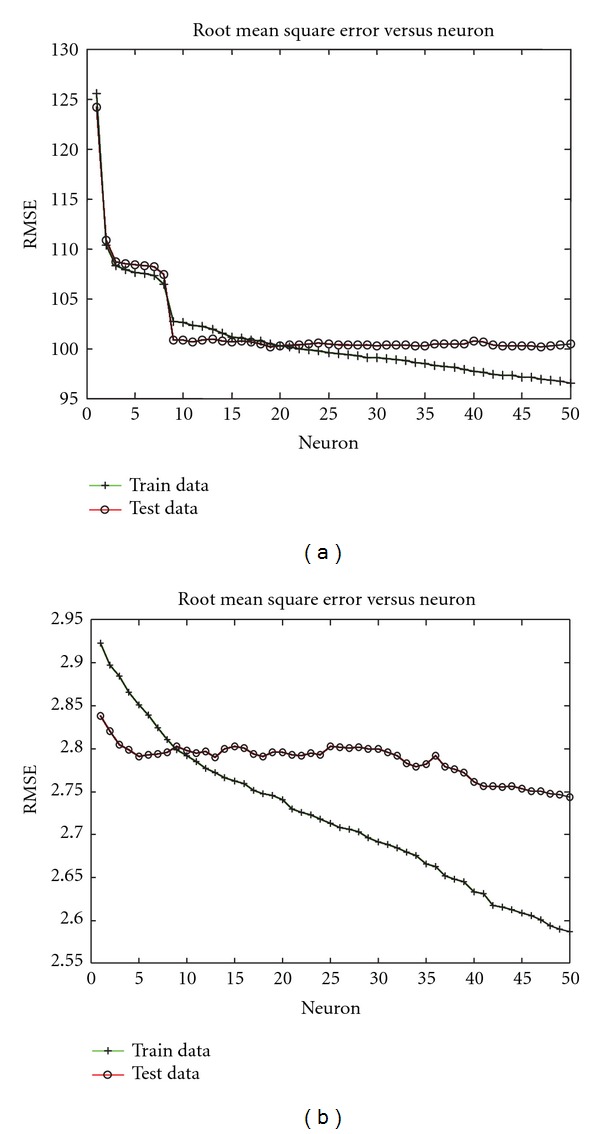
Root mean square error (RMSE) as a function of number of neurons in the multiple-trait neuro-fuzzy models: (a) prediction of milk yield EBV in experiment 20, and (b) prediction of fat yield EBV in experiment 20.

Figures 2 and 3 show the relationship between the number of neurons in the NFS analyses and the root mean square error of prediction in the training and testing sets. These figures clearly illustrate the danger of overfitting the training data. In every case, increasing model complexity (via the addition of more neurons) continuously improved predictive ability within the training set. However, predictive ability within the testing set, which is the true measure of expected performance in future, independent data sets, can be compromised by overfitting. In some cases (e.g., Figures 2(a) and 3(a)) the cost of overfitting was small, but in other cases (e.g., Figure 2(b)) performance in the testing set was significantly impaired by unnecessary increases in model complexity. In practice, users should monitor cross-validation predictive ability in the tuning set (i.e., a “set aside” portion of the training set) to avoid overfitting and thereby optimize parameters of the model. 

## 4. Conclusions

The current methods for computing EBV, which involve simultaneous animal model BLUP analysis of all performance-recorded animals in the population, are computationally intensive and time-consuming. As such, EBVs are computed only periodically, usually two or three times per year. Therefore, it may be useful to develop an alternative approach for routine computation of EBV of dairy sires and cows, so that new data can be incorporated as soon as it becomes available. With this in mind, we evaluated the possibility of calculating approximate EBV using computationally efficient algorithms from the fields of artificial intelligence and machine learning, namely, artificial neural networks, or ANN, and neuro-fuzzy systems, or NFS. Using ANN and NFS approaches, we produced single trait predictions of milk yield EBV that had correlations of 0.917 and 0.926, respectively, and for fat yield EBV that had correlations of 0.926 and 0.932, respectively, with reference EBV. Furthermore, joint prediction of milk and fat yield EBV in multiple-trait implementations of ANN provided correlations of 0.925 and 0.930, respectively, with reference EBV for milk and fat production. The same prediction with NFS provided a correlation of 0.935 and 0.949 with reference EBV, respectively, for milk and fat. 

In most cases, NFS tended to provide greater predictive ability than ANN. However, the difference in performance between these two methods was rather small. For both methods, increasing the number of input variables led to predictions of EBV with greater accuracy. In general, however, the NFS approach seemed to provide slightly more consistent results, and this method may be more robust to “noise” in specific data sets or redundancies among specific combinations of explanatory variables. Some novel aspects of the NFS approach are advantageous as compared with conventional ANN methodology. For example, learning of model trees in the LOLIMOT algorithm leads to automatic adaptation of the complexity of the network structure to the requirements of a particular application. Considerable post-pruning would be required to achieve similar results using ANN. In addition, every neuron in the NFS implementation, via the LOLIMOT algorithm, is a linear regressor, and therefore the resulting solution is much more transparent than that of an ANN. In addition to not having learning problems such as suboptimality due to local minima, it can provide a better explanation engine and means to use partial expert knowledge in the linear form.

 Lastly, it must be emphasized that the application of such novel methods for computation of EBV in animal breeding is quite new, and as such a period of learning and adaptation will be required before such approaches can be implemented in an optimal manner. 

## Figures and Tables

**Table 1:** Inputs and outputs of various twenty experiments in this study.

Experiment no.	1	2	3	4	5	6	7	8	9	10	11	12	13	14	15	16	17	18	19	20
Inputs																				
Age	∗	∗	∗	∗	∗	∗	∗	∗	∗	∗	∗	∗	∗	∗	∗	∗	∗	∗	∗	∗
Days in milk		∗	∗		∗	∗		∗	∗	∗	∗	∗	∗	∗	∗	∗	∗	∗	∗	∗
Milk 2x	∗	∗	∗	∗	∗	∗	∗	∗	∗	∗	∗	∗	∗	∗	∗	∗	∗	∗	∗	∗
Fat 2x										∗	∗	∗	∗	∗	∗	∗	∗	∗	∗	∗
Herd mean milk 2x										∗	∗	∗	∗	∗	∗	∗	∗	∗	∗	∗
Herd mean fat 2x										∗	∗	∗	∗	∗	∗	∗	∗	∗	∗	∗
Herd mean milk total																				∗
Total milk																	∗		∗	∗
Temperature			∗			∗			∗	∗	∗	∗	∗	∗	∗	∗	∗	∗	∗	∗
Humidity										∗	∗	∗	∗	∗	∗	∗	∗	∗	∗	∗
Day length										∗	∗	∗	∗	∗	∗	∗	∗	∗	∗	∗
Milk EBV of dam	∗	∗	∗				∗	∗	∗	∗	∗	∗	∗	∗	∗	∗	∗	∗	∗	∗
Fat EBV of dam											∗		∗	∗	∗	∗	∗	∗	∗	∗
Milk EBV of sire				∗	∗	∗	∗	∗	∗			∗	∗					∗	∗	∗
Fat EBV of sire															∗			∗	∗	∗
Outputs																				
Milk EBV	∗	∗	∗	∗	∗	∗	∗	∗	∗	∗	∗	∗	∗			∗	∗	∗	∗	∗
Fat EBV														∗	∗	∗	∗	∗	∗	∗

**Table 2:** Mean square error, root mean square error, and correlation in thirteen MLP and neuro-fuzzy networks for predicting milk EBV.

Networks		MLP		LOLIMOT	
Error criteria		RMSE	*r*	RMSE	*r*
Experiment no.	1	192.3	0.69	184.022	0.81
	2	156.5	0.81	154.5	0.82
	3	149.8	0.83	153.4	0.83
	4	208.1	0.63	210.6	0.63
	5	212.0	0.61	206.8	0.66
	6	172.8	0.67	205.5	0.68
	7	154.1	0.82	144.2	0.82
	8	151.6	0.82	143.1	0.83
	9	144.3	0.85	143.4	0.84
	10	109.7	0.91	113.1	0.92
	11	117.9	0.90	113.0	0.92
	**12**	106.7	0.92	**101.8**	**0.93**
	**13**	**106.2**	**0.92**	102.0	0.93

**Table 3:** Mean square error, root mean square error, and correlation in two MLP and neuro-fuzzy networks for predicting fat EBV.

Networks		MLP		LOLIMOT	
Error criteria		RMSE	*r*	RMSE	*r*
Experiment no.	14	3.1	0.91	3.3	0.91
	**15**	**2.7**	**0.93**	**2.8**	**0.93**

**Table 4:** Mean square error, root mean square error, and correlation in five MLP and neuro-fuzzy networks for predicting milk and fat EBV simultaneously.

Networks		MLP				LOLIMOT			
Trait		Milk		Fat		Milk		Fat	
Error criteria		RMSE	*r*	RMSE	*r*	RMSE	*r*	RMSE	*r*
	16	122.3	0.89	4.45	0.88	113.1	0.92	3.30	0.91
	17	117.7	0.90	4.33	0.88	113.7	0.92	3.32	0.91
Experiment no.	18	105.5	0.90	5.11	0.92	102.6	0.93	2.84	0.94
	19	103.8	0.92	5.07	0.92	102.4	0.93	2.77	0.94
	**20**	**101.4**	**0.93**	**4.93**	**0.93**	**100.2**	**0.94**	**2.75**	**0.95**

## Abbreviations

ANN: Artificial neural network
MLP: Multilayer perceptron
LOLIMOT: Locally linear model tree
LLNF: Locally linear neuro-fuzzy
NFS: Neuro fuzzy system
EBV: Estimated breeding value
MF: Membership function.
